# GCP16 stabilizes the DHHC9 subfamily of protein acyltransferases through a conserved C-terminal cysteine motif

**DOI:** 10.3389/fphys.2023.1167094

**Published:** 2023-03-23

**Authors:** Phillip L. Nguyen, Wendy K. Greentree, Toshimitsu Kawate, Maurine E. Linder

**Affiliations:** Department of Molecular Medicine, Cornell University, Ithaca, NY, United States

**Keywords:** post-translational modification (PTM), protein acylation, protein stability, protein com-plex, membrane protein, Golga7b, GOLGA7

## Abstract

Protein *S*-acylation is a reversible lipid post-translational modification that allows dynamic regulation of processes such as protein stability, membrane association, and localization. Palmitoyltransferase ZDHHC9 (DHHC9) is one of the 23 human DHHC acyltransferases that catalyze protein *S*-acylation. Dysregulation of DHHC9 is associated with X-linked intellectual disability and increased epilepsy risk. Interestingly, activation of DHHC9 requires an accessory protein—GCP16. However, the exact role of GCP16 and the prevalence of a requirement for accessory proteins among other DHHC proteins remain unclear. Here, we report that one role of GCP16 is to stabilize DHHC9 by preventing its aggregation through formation of a protein complex. Using a combination of size-exclusion chromatography and palmitoyl acyltransferase assays, we demonstrate that only properly folded DHHC9-GCP16 complex is enzymatically active *in vitro*. Additionally, the ZDHHC9 mutations linked to X-linked intellectual disability result in reduced protein stability and DHHC9-GCP16 complex formation. Notably, we discovered that the C-terminal cysteine motif (CCM) that is conserved among the DHHC9 subfamily (DHHC14, -18, -5, and -8) is required for DHHC9 and GCP16 complex formation and activity *in vitro*. Co-expression of GCP16 with DHHCs containing the CCM improves DHHC protein stability. Like DHHC9, DHHC14 and DHHC18 require GCP16 for their enzymatic activity. Furthermore, GOLGA7B, an accessory protein with 75% sequence identity to GCP16, improves protein stability of DHHC5 and DHHC8, but not the other members of the DHHC9 subfamily, suggesting selectivity in accessory protein interactions. Our study supports a broader role for GCP16 and GOLGA7B in the function of human DHHCs.

## Introduction

Cells utilize a wide array of protein post-translational modifications to extend the chemical properties of the 20 standard amino acids, dramatically expanding the ways in which proteins regulate cellular processes. In eukaryotes, protein *S*-acylation is the process in which long-chain fatty acids (with palmitate being the most prevalent) are added to proteins at cysteine residues *via* a labile thioester linkage. While the fundamental chemical effect of *S*-acylation is a local increase in protein hydrophobicity, the reversible nature of *S*-acylation can result in a range of consequences such as dynamic changes in protein stability, membrane affinity and binding, and protein trafficking and localization (Chamberlain and Shipston, 2015).

DHHC protein acyltransferases (PATs) are the enzymes responsible for catalyzing the addition of long-chain fatty acids to substrate proteins (Gottlieb and Linder, 2017). Members of the DHHC family are conserved throughout eukaryotic evolution, with 5-7 members in yeast and as many as 23 members in humans (Mitchell et al., 2006; Zhang et al., 2013). Furthermore, dysregulation of DHHC proteins is associated with a myriad of diseases that include cancers and neurodegenerative disorders (Young et al., 2012; Yeste-Velasco et al., 2015). DHHC proteins are aptly named for the conserved Asp-His-His-Cys motif required for their PAT activity, which is embedded in a cysteine-rich domain (CRD) involved in zinc ion binding (Gonzalez Montoro et al., 2013; Gottlieb et al., 2015; Rana et al., 2018). Additionally, all DHHC proteins are multipass transmembrane proteins, with subfamily diversity stemming from varied membrane topologies, sequence divergence in the amino- and carboxy-terminal regions, and protein partner requirements (Lobo et al., 2002; Swarthout et al., 2005; Salaun et al., 2020). It is generally accepted that DHHC proteins utilize a two-step ping-pong mechanism (Mitchell et al., 2010; Jennings and Linder, 2012). First, the DHHC protein uses acyl-CoA as an acyl group donor to form an acyl-enzyme intermediate; then, the acyl group is transferred from the DHHC cysteine to the target cysteine on the protein substrate. While this mechanism is thought to be shared by all DHHCs, a majority of mechanistic and structural insight has been determined from studies on one subfamily of DHHCs that include DHHC2, 3, and 20 (Jennings and Linder, 2012; Gottlieb et al., 2015; Rana et al., 2018). Thus, this leaves many other DHHC proteins underrepresented, and potentially subfamily-specific insights are yet to be discovered.

In humans, DHHC9 was the first DHHC protein discovered to require an accessory protein—GCP16 (also known as GOLGA7)—for its enzymatic function, based on sequence homology with the yeast Ras PAT Erf2-Erf4 (Swarthout et al., 2005). DHHC9 colocalizes with and requires GCP16 for its enzymatic activity (Swarthout et al., 2005). GCP16 is a small peripheral membrane protein that is itself palmitoylated, and it associates with proteins involved in vesicular transport at the Golgi (Ohta et al., 2003). However, it is unclear what the exact role of GCP16 is in DHHC9 protein regulation, how GCP16 supports DHHC PAT activity, and to what extent GCP16 or a related protein GOLGA7B function within the DHHC protein family. Furthermore, loss of function mutations in ZDHHC9 result in X-linked intellectual disability (XLID), with affected individuals displaying neurodevelopmental delay, seizures, and facial dysmorphism (Raymond et al., 2007; Baker et al., 2015; Han et al., 2017; Schirwani et al., 2018). Despite the strong clinical relevance, the molecular basis for how ZDHHC9 mutations affect DHHC9 protein function and regulation is incompletely understood. In this study, we sought to better understand the role of GCP16 in the DHHC9-GCP16 PAT complex, and we investigated the stability of the DHHC9 disease mutants. We also assessed whether the accessory proteins GCP16 and GOLG7B associate more widely with members of the DHHC protein family.

## Materials and methods

### Construction of expression plasmids

Amino acid sequences were obtained from the UniProt database for the following human proteins: DHHC9 (Q9Y397), DHHC14 (Q8IZN3), DHHC18 (Q9NUE0), DHHC5 (Q8VDZ4), DHHC8 (Q9ULC8), DHHC3 (Q9NYG2), DHHC20 (Q5W0Z9), and GCP16 (Q7F5G4). Genes were synthesized based on protein sequences (GenScript, Piscataway, NJ). The plasmid encoding GOLGA7B was obtained from the Harvard Medical School Plasmid repository. All DHHC genes used were PCR amplified to exclude the start methionine and to include flanking BamHI/XhoI sites. Similarly, GOLGA7 and GOLGA7B were amplified to exclude the start methionine and to include AgeI/NotI sites. All genes were subcloned *via* standard molecular biology techniques into their respective expression vectors. Any mutations were generated *via* quick change PCR.

For FSEC, we used a dual mammalian expression vector modified from the pIRES-EGFP RK6 vector provided by M. Mayer, National Institutes of Health, Bethesda, MD. The vector encoded a start methionine followed by mNeonGreen (A0A1S4NYF2), a BamHI/XhoI insertion site, an EMCV internal ribosomal entry site (IRES), an AgeI/NotI insertion site, and a stop codon. DHHC genes were incorporated using BamHI/XhoI, and GOLGA7 or GOLGA7B genes were incorporated using AgeI/NotI.

For protein purification, genes were subcloned into a modified pFastBac baculovirus expression vector (Thermo Fisher Scientific, Waltham, MA). DHHC9, DHHC9 TM (C283S, C284S, and C288S), and GCP16 were encoded to express a C-terminal strep-tag. DHHC14 and DHHC18 were encoded to express an N-terminal strep-tag.

### Fluorescence-detection size-exclusion chromatography

Human embryonic kidney (HEK) cells were maintained in DMEM medium (Thermo Fisher Scientific) supplemented with 10% fetal bovine serum (Atlanta Biologicals, Flowery Branch, GA), and 10 μg/ml gentamicin (Thermo Fisher Scientific) per manufacturer’s instructions. For FSEC experiments, cells were transfected at 80–95% confluency with 2.5 µg of expression plasmid using jetPRIME transfection reagent per the manufacturer’s instructions. Cells were grown at 37°C for 48 h. The media was aspirated, cells were suspended and washed in 2 ml of cold PBS, and samples were lysed in 150 µL of lysis buffer (1x PBS, 1% n-Dodecyl-β-D-Maltopyranoside (DDM), 1x Roche protease inhibitor) for 30 min at 4 °C with rotation. Whole-cell lysates were cleared by centrifugation in an Eppendorf FA-45–24-11 rotor at 21,000 x g for 10 min at 4°C. The supernatant was further cleared by ultracentrifugation in a TLA 100.3 rotor at 265,000 x g for 20 min. An aliquot (50 µL) was applied onto a Superdex 200 Increase 10/300 GL column pre-equilibrated with running buffer (1x PBS, 0.5 mM DDM). The eluate from the SEC column was passed through a fluorometer set to excitation, 480nm, and emission, 508 nm.

For quantitation, peak intensities in arbitrary fluorescence units were obtained at the expected DHHC peak for every profile. For DHHC9 constructs, values were normalized to either DHHC9 or DHHC9 N2C1, depending on the set of constructs tested for that given day. Data sets across different days were then normalized to DHHC9 globally. For experiments that included other DHHCs, i.e., DHHC14, 18, 5, 8, 3, and 20, raw intensity values were used without normalization. For statistical analysis, we used two-tailed t-tests assuming unequal variances against the null-hypothesis that GCP16/GOLGA7B co-expression has no significant effect on DHHC protein signal.

### Expression and purification of DHHC proteins and DHHC-GCP16 complexes

All constructs were expressed using the Invitrogen Bac-to-Bac^®^ baculovirus-insect cell expression system. Sf9 cells (1–2L) were infected at 2.5—4.0 × 10^6^ cells/mL with P2 virus (107–108 PFU/ml, 30 ml/L). Cells were incubated at 27°C for 24 h and moved to 18 °C for an additional 48 h. All purification steps were done on ice or with 4°C buffer unless stated otherwise. Cells were harvested by centrifugation at 2,040 x g and washed with 200 ml of PBS. Cells were solubilized in buffer A (PBS, 15% glycerol, 0.5 mM TCEP, 0.5ug/mL leupeptin, 2ug/mL aprotinin, 0.5ug/mL pepstatin A, and 0.5 mM phenylmethylsulfonyl fluoride) containing 1% DDM w/v at a ratio of 250 mg DDM/g of cells. Solubilization was performed at 4 °C with stirring for 1 hour. Large debris were removed by centrifugation at 12,000 x g. The supernatant was further clarified by ultracentrifugation at 185,000 x g for 45 min to remove remaining insolubilized material. To the supernatant, 2 ml of equilibrated StrepTactin Sepharose High Performance resin (GE Healthcare, Marlborough, MA) was incubated with stirring for 1-h. The resin was collected by centrifugation and transferred to a gravity column (Bio-Rad). The resin was washed with 10 resin bed volumes of wash buffer (buffer A with 0.5 mM DDM) and eluted with elution buffer (buffer A with 2.5 mM desthiobiotin, 0.5 mM DDM). Protein was concentrated using Amicon ultra concentrators. Size exclusion chromatography was done using a Superdex 200 (GE Healthcare) in SEC buffer (150 mM NaCl, 20 mM HEPES pH 7.4, 0.5 mM TCEP, 15% glycerol, 0.5 mM DDM).

### PAT assays

PAT assays were performed immediately following purification and SEC as described previously (Swarthout et al., 2005). Protein concentrations were determined by NanoDrop (Thermo Scientific) using predicted extinction coefficients. Enzyme was diluted to 250 nM in enzyme dilution buffer (50 mM MES pH 6.4, 100 mM NaCl, 10% glycerol, 0.05% DDM, 0.5 mM TCEP). H-Ras (80 μM, purified from insect cells infected with recombinant Baculovirus) was diluted to 10 µM in enzyme dilution buffer. For a 50 µL reaction, the final concentration was 50 nM enzyme, 2 µM H-Ras, 1 µM [^3^H]-palm-CoA. Each reaction was incubated at room temperature for 10 min. Reactions were quenched in SDS loading dye containing 25 mM TCEP. Samples were split and run on two SDS-PAGE gels in parallel. For fluorography, one gel was exposed to a solution of 1M sodium salicylate, 15% methanol, dried under vacuum for 2 h, and exposed to film for 4–15 days. For liquid scintillation spectroscopy, the gel was stained with Coomassie, H-Ras bands were extracted, and a section of gel around the expected DHHC protein size was extracted. Extracted gel pieces were dissolved in 500 µL Soluene overnight at 37°C. Ultima Gold scintillation fluid (5 ml) was added, and disintegrations per minute (DPM) was measured using a scintillation counter after allowing for stabilization for 8–24 h. For each experiment, we used DHHC20 as a positive control and H-Ras without enzyme to account for non-enzymatic acylation. Background DPM was subtracted from each sample before converting counts to picomoles based on the specific activity of palmitoyl-CoA.

## Results

### GCP16 stabilizes DHHC9 through a conserved C-terminal cysteine motif

**FIGURE 1:**
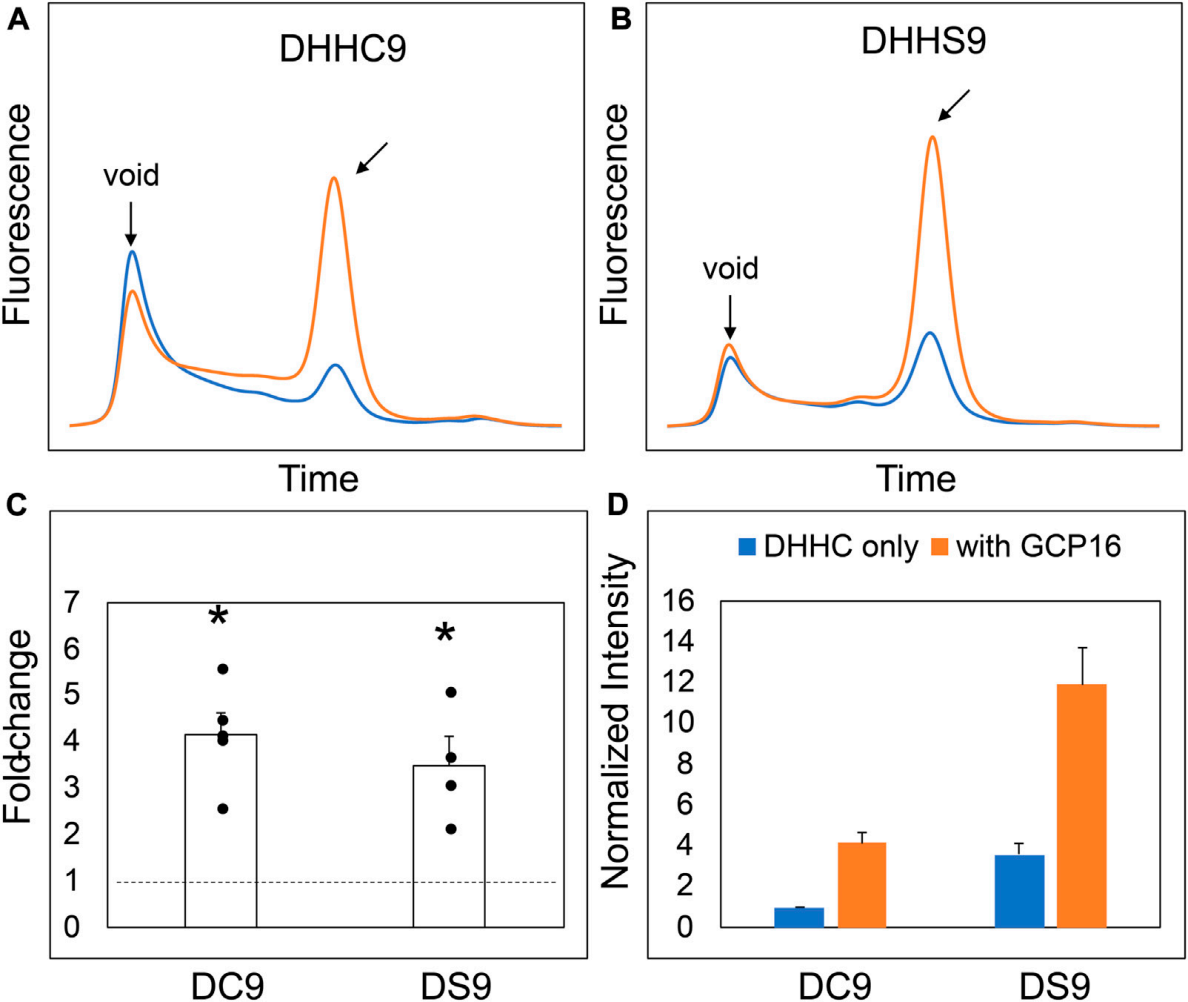
DHHC9 is Stabilized by Co-expression with GCP16 Independent of the Catalytic Cysteine. FSEC profiles of **(A)** DHHC9 and **(B)** DHHS9. HEK cells were transfected with vector to express the indicated constructs without or with GCP16 co-expression shown with blue and orange traces, respectively. Cells were solubilized in a DDM-containing buffer, and the cleared lysate was analyzed *via* SEC detected by fluorescence at excitation and emission wavelengths of 480 nm and 508 nm, respectively. Arrows indicate the void retention time and the approximate retention time corresponding to the size of DHHC9. Bar charts showing **(C)** average fold-change and **(D)** normalized max intensity upon GCP16 co-expression for the indicated constructs at n ≥ 4 experiments. Normalization was done as described in the experimental procedures. The dashed line indicates a fold-change of 1. Error bars represent the standard error of the mean. Asterisks indicate a significance for *p*-value <0.05 determined by two-tailed *t*-test against the null hypothesis.

Our initial attempts to express and purify DHHC9 from Sf9 cells were met with low protein yield, reduced purity, and extensive protein aggregation. This was consistent with a previous report that DHHC9 purified without GCP16 resulted in an increased fraction of DHHC9 protein being proteolyzed (Swarthout et al., 2005). Thus, we reasoned that one function of GCP16 is to stabilize DHHC9 and prevent misfolding or aggregation. We used fluorescence-detection size-exclusion chromatography (FSEC) to examine the effect of GCP16 on DHHC9 protein stability in cell lysates (Kawate and Gouaux, 2006). DHHC9 fused with N-terminal mNeonGreen was expressed in HEK293 cells with or without GCP16. We chose to fuse the fluorescent protein to the DHHC9 N-terminus to minimize perturbing its C-terminus, based on previous reports that N-terminal modifications to Erf2 had no effect on its stability and that the C-terminus of DHHC proteins are generally thought to mediate protein-protein interactions (Mitchell et al., 2012; Malgapo and Linder, 2021). Cells were solubilized in a dodecylmaltoside (DDM)-containing buffer, and cleared lysates were applied to a gel-filtration column coupled to a fluorometer to monitor fluorescence (Figure 1). DHHC9 expressed alone showed a prominent void peak characteristic of high-molecular weight protein aggregate and only a minor peak at the expected retention time for a well-folded, monomeric protein (Figure 1A blue trace). By contrast, DHHC9 co-expressed with GCP16 showed improved monodispersity and a 4-fold increase in magnitude (Figure 1A orange trace, C, D). To determine if the GCP16-mediated stabilizing effect is dependent on the catalytic cysteine, we performed the same assay using DHHS9, in which the catalytic cysteine was mutated to serine. Compared to DHHS9 alone, DHHS9 co-expressed with GCP16 showed an almost identical improvement in monodispersity and expression as DHHC9 (Figures 1B–D). These data suggest that DHHC9 is stabilized by GCP16 co-expression, and this effect is not dependent on DHHC9 catalytic activity.

**FIGURE 2:**
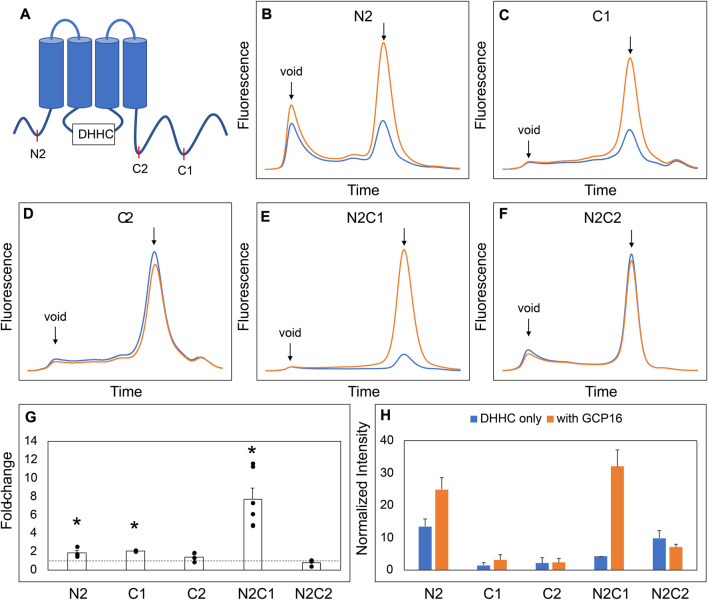
A Conserved Region in the DHHC9 CTD is Required for GCP16-mediated Stabilization. **(A)** Cartoon depicting the tested DHHC9 truncations. **(B–F)** FSEC profiles for the indicated constructs. HEK cells were transfected with DHHC9 without (blue) or with GCP16 (orange). Cells were solubilized in a DDM-containing buffer, and the cleared lysate was analyzed by FSEC. Bar charts showing **(G)** average fold-change and **(H)** normalized max intensity upon GCP16 co-expression for the indicated constructs at n ≥ 3 experiments. Error bars represent the standard error of the mean. Asterisks indicate a significance for *p*-value <0.05 determined by two-tailed *t*-test against the null hypothesis.

Next, we asked which regions and amino acid residues of DHHC9 are required for the GCP16-mediated stabilizing effect. We used evolutionary conservation to generate combinatorial N- and C-terminal deletions of DHHC9 (Table 1; Supplementary Figure S1) and assessed the effect of GCP16 co-expression for each construct by FSEC (Figure 2). For DHHC9 with N-terminal truncation N2, C-terminal truncation C1, or both, GCP16 co-expression improved protein behavior, similar to the full-length construct (Figures 2B,C,E,G,H). However, for DHHC9 constructs containing truncation C2, GCP16 co-expression showed no significant difference when compared to DHHC9 alone (Figures 2D,F). We observed that some individual truncations alter total levels of DHHC9 detected (Figure 2H). For example, constructs N2 and N2C1 improved DHHC9 protein stability in the absence of GCP16, possibly by removing disordered/aggregation-prone regions of DHHC9 (Figures 2B,E,G,H). Non-etheless, the addition of GCP16 further increased the stabilizing-effect in constructs that included truncation C1. Thus, DHHC9 residues between truncation C1 and C2 are required for GCP16-mediated stabilization.

**TABLE 1:** DHHC9 Constructs. Table denoting the generated constructs (left) and amino acid changes (right).

Construct	Description
DHHC9 (full-length)	
DHHC9 N2	Δ2-19
DHHC9 C1	Δ301-364
DHHC9 C2	Δ278-364
DHHC9 N2C1	Δ2-19, Δ301-364
DHHC9 N2C1.1	Δ2-19, Δ295-364
DHHC N2C29 N2C1.2	Δ2-19, Δ289-364
DHHC9 N2C1.3	Δ2-19, Δ282-364
DHHC9	Δ2-19, Δ278-364

**FIGURE 3:**
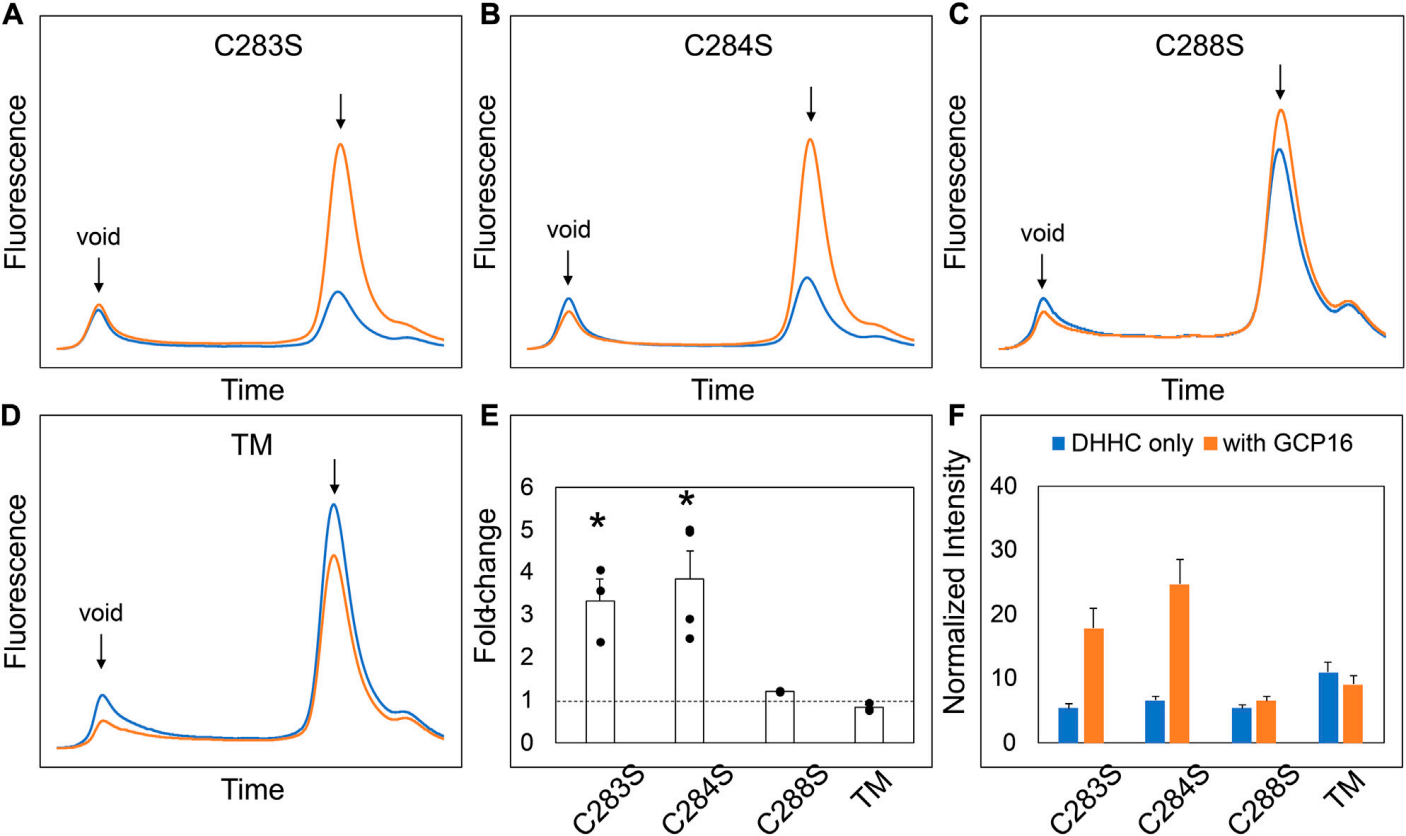
Cysteines in the DHHC9 CTD are required for GCP16-mediated Stabilization. **(A–C)** FSEC profiles for the indicated cysteine to serine mutation and **(D)** the triple mutation (TM) in the DHHS9 N2C1 parent construct. HEK cells were transfected with DHHC9 without (blue) or with GCP16 (orange). Cells were solubilized in DDM-containing buffer, and the cleared lysate was analyzed *via* FSEC. Bar charts showing **(E)** average fold-change and **(F)** normalized max intensity upon GCP16 co-expression for the indicated constructs at n ≥ 3 experiments. The dashed line indicates a fold-change of 1. Error bars represent the standard error of the mean. Asterisks indicate a significance for *p*-value <0.05 determined by two-tailed *t*-test against the null hypothesis.

We next sought to determine the exact residues between DHHC9 C1 and C2 that are required for GCP16-mediated stabilization. We made finer truncations between regions C1 and C2 in the N2C1 background and assayed for the effect of GCP16 co-expression using FSEC (Supplementary Figure S2). Much like the parent construct, truncations C1.1 and C1.2 showed an increase in expression and monodispersity with GCP16 co-expression (Supplementary Figures S2B, S2C, S2E, S2F). However, truncation C1.3 displayed no significant difference whether GCP16 was co-expressed or not (Supplementary Figure S2D, S2E, S2F). Between truncations C1.2 and C1.3, we identified a conserved sequence “CCXXXC” at residues 283–288, which we refer to as the C-terminal cysteine motif (CCM). Given that cysteines may play a role in protein-protein interactions, we tested whether mutation of these CCM cysteines would abolish the stabilizing effect. We generated serine mutants at each individual cysteine in the CCM using the DHHS9 N2C1 background and assessed the effect of GCP16 co-expression on DHHC9 protein (Figure 3). DHHS9 NC21 was used as the parent construct because it gave a more consistent FSEC signal in the absence of GCP16 than that of full-length DHHC9. Mutations C283S and C284S showed a diminished but statistically significant improvement in protein expression when co-expressed with GCP16 (Figures 3A,B,E,F). Interestingly, C288S or the triple mutations at all three cysteines (TM) showed no effect of GCP16 co-expression on DHHC9 protein quality (Figures 3C–F). Taken together, these data show that DHHC9 protein expression and monodispersity is significantly improved by GCP16 co-expression, and this stabilization effect requires a conserved DHHC9 C-terminal cysteine motif, with cysteine 288 being the most critical.

### DHHC9 CCM is required for complex formation with GCP16

**FIGURE 4:**
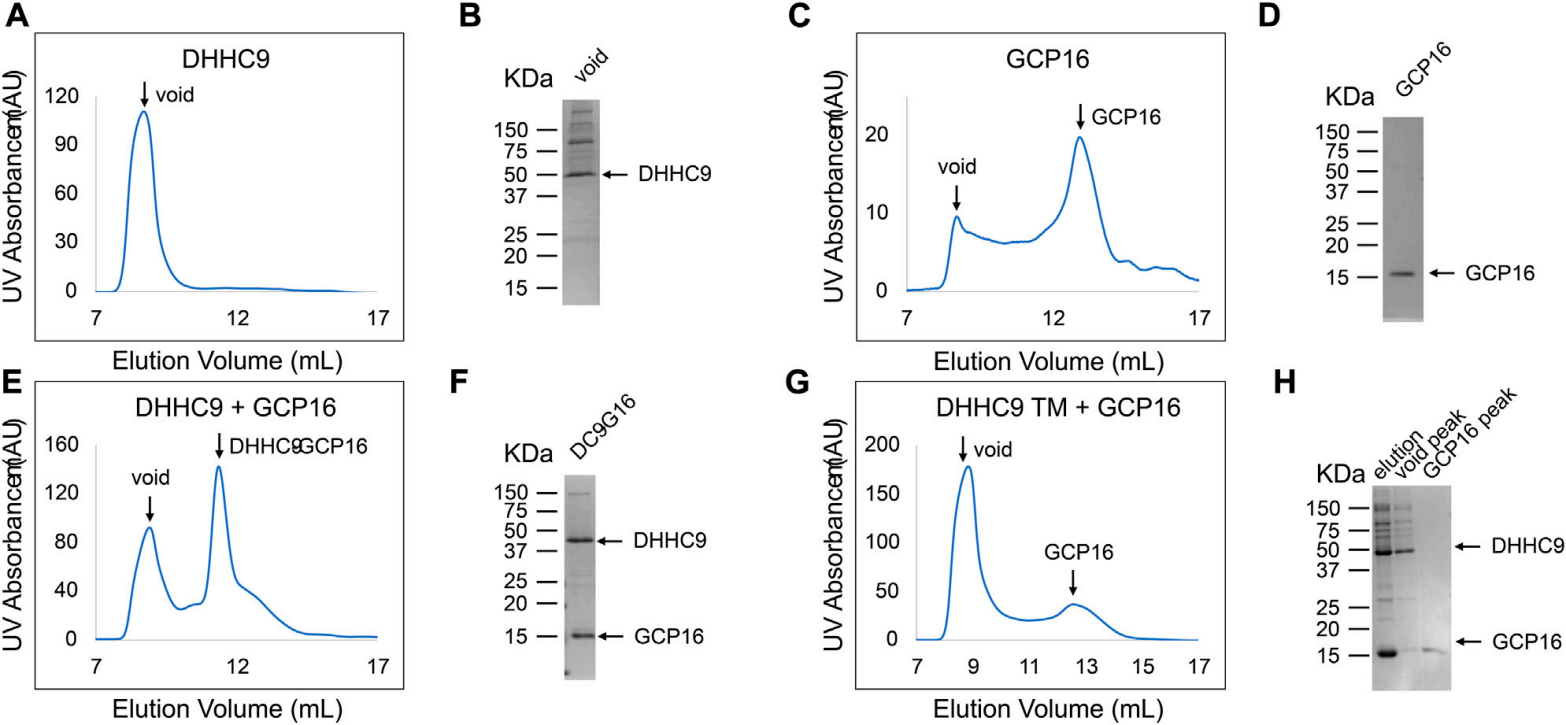
DHHC9 CTD Cysteines are Required for Complex Formation with GCP16. Sf9 cells were infected with recombinant baculovirus encoding the indicated variations of the following: DHHC9-strep, DHHC9 with the conserved triple cysteines mutated to serine (DHHC9 TM), or GCP16-strep. Protein was purified *via* strep-affinity purification and SEC as in the experimental procedures. SEC profiles and SDS-PAGE Coomassie analyses for **(A,B)** DHHC9 **(C,D)** GCP16, **(E,F)** the DHHC9-GCP16 complex, and DHHC9-TM co-purified with GCP16 **(G,H)**. Arrows in the SEC profiles correspond to the labeled lanes for the corresponding SDS-PAGE.

While FSEC is an efficient way to probe whether GCP16 expression influences DHHC9 stability, it cannot address whether the two proteins are interacting. To determine if DHHC9 and GCP16 form a complex, we co-expressed these two proteins in Sf9 insect cells, affinity purified, and assessed whether they co-elute in SEC (Figure 4). Across multiple experiments, affinity-purified DHHC9 in the absence of GCP16 consistently eluted at the void volume (Figure 4A). This and the observation that the protein remains impure after tandem affinity chromatography and SEC (Figure 4B), suggested that purified DHHC9 primarily exists as a high molecular weight protein aggregate. GCP16 alone eluted around ∼13ml, which was consistent with its molecular size with detergent micelle (Figures 4C, D). When DHHC9 and GCP16 were co-expressed, both proteins co-eluted at a volume of ∼11 ml (Figures 4E, F) with a monodispersed peak, supporting that DHHC9-GCP16 exists as a folded complex.

Next, we tested whether the CCM of DHHC9 is required for complex formation with GCP16. We co-purified DHHC9 TM with GCP16 and analyzed the SEC profile. We confirmed by SDS-PAGE that both DHHC9 TM and GCP16 were present in the elution from affinity purification (Figure 4H lane 1). However, the SEC profile exhibited two distinct peaks at the void volume and at ∼13 ml (Figure 4G), paralleling what was seen for DHHC9 and GCP16 when purified separately. Furthermore, SDS-PAGE analysis showed enrichment for DHHC9-strep in the fraction corresponding to the void peak and GCP16-strep in the fraction at ∼13 ml (Figure 4H lane 2–3). Together, these experiments support that DHHC9 and GCP16 form a complex when co-expressed and co-purified. Furthermore, mutation of the conserved DHHC9 C-terminal cysteines prevents complex formation under these conditions of detergent solubilization.

### DHHC9 enzymatic activity for H-Ras correlates with protein folding

**FIGURE 5:**
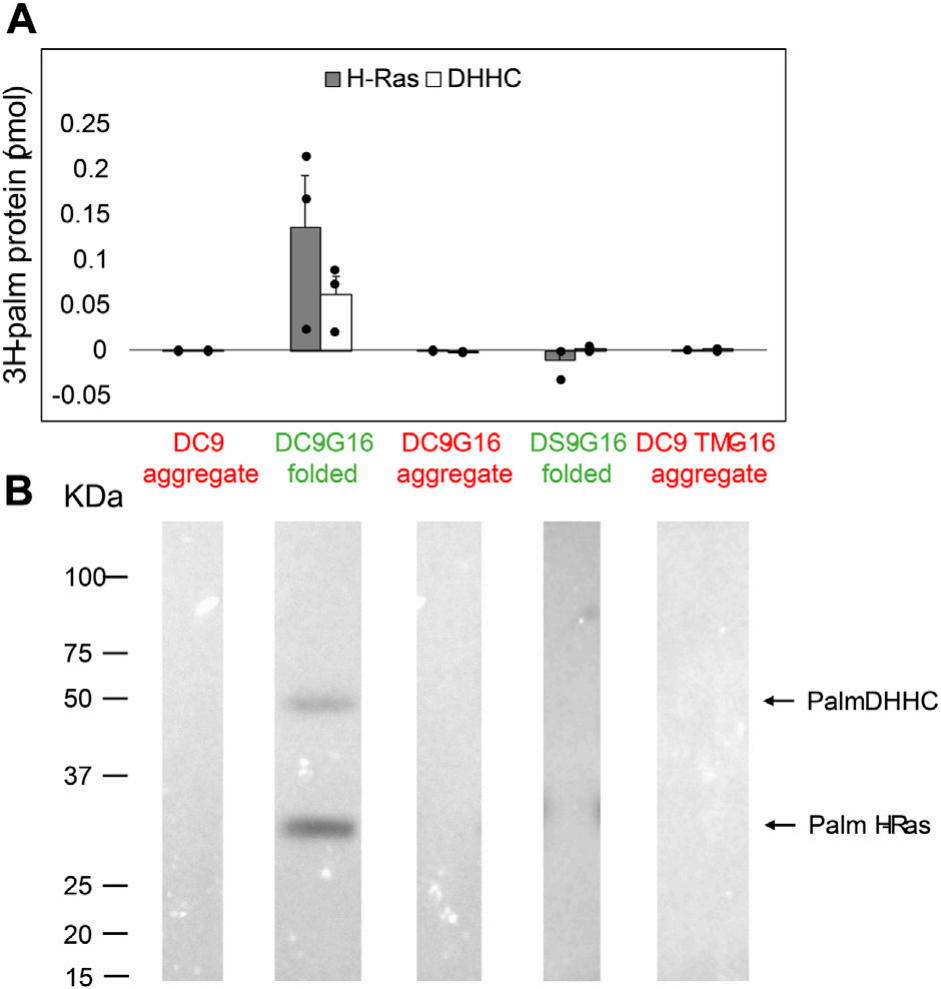
DHHC9 Requires Proper Protein Folding for H-Ras Activity. DHHC9 constructs were purified and classified into aggregated (red) or folded (green) states based on their respective SEC profiles. Purified DHHC9 was assayed for activity using H-Ras and [^3^H]-palmitoyl CoA. Radiolabeled protein was quantified *via* liquid scintillation spectroscopy **(A)** and detected *via* fluorography **(B)**. The *Y*-axis represents picomoles of ^3^H-labeled protein after subtracting background radiation. Grey bars represent the quantity of labeled H-Ras, and white bars represent the quantity of labeled DHHC protein. Error bars represent the standard error of the mean for n ≥ 2 independent protein preparations. Arrows indicate bands corresponding to radiolabeled DHHC9 and H-Ras.

Protein *S*-acylation by DHHC proteins occurs *via* a two-step mechanism (Mitchell et al., 2010; Jennings and Linder, 2012). The DHHC protein uses acyl-CoA to form an acyl-enzyme intermediate; then, upon substrate binding, the acyl group is transferred to the target cysteine on the protein substrate. We previously demonstrated that GCP16 increases the equilibrium levels of both autoacylated DHHC9 and its substrate H-Ras *in vitro* (Swarthout et al., 2005). However, it remains unclear whether GCP16 directly affects catalysis, or whether GCP16 simply increases the amount of folded and active DHHC9. Therefore, we purified DHHC9 with or without GCP16 and classified whether the protein was folded or aggregated based on SEC profiles. Immediately following purification and SEC, we assayed PAT activity using H-Ras and [^3^H]-Palmitoyl-CoA. The folded DHHC9-GCP16 complex exhibited radiolabeling for both H-Ras and autoacylated DHHC9 (Figure 5 lane 2). The observed activity was attributed to the catalytic activity of DHHC9, as catalytically inactive DHHS9 protein failed to show any PAT activity (Figure 5 lane 4). In contrast, the aggregated DHHC9-GCP16 did not exhibit detectable PAT activity (Figure 5 lane 3). Likewise, aggregated DHHC9 purified in the absence of GCP16 exhibited no detectable activity (Figure 5 lane 1). Similarly, DHHC9 TM was unable to form a complex with GCP16 and resulted in only aggregated protein. This aggregated DHHC9 TM exhibited no detectable activity for H-Ras (Figure 5 lane 5). Taken together, these experiments suggest that DHHC9 requires GCP16 for its enzymatic activity. DHHC9 co-purified with GCP16 results in enrichment of a folded DHHC9 state, which is enzymatically active. However, when GCP16 is absent or when DHHC9 is unable to form a complex with GCP16 (as is the case with DHHC9 TM), essentially all DHHC9 protein is aggregated and exhibits no detectable PAT activity for H-Ras.

### DHHC9 disease mutations result in reduced protein stability

**FIGURE 6:**
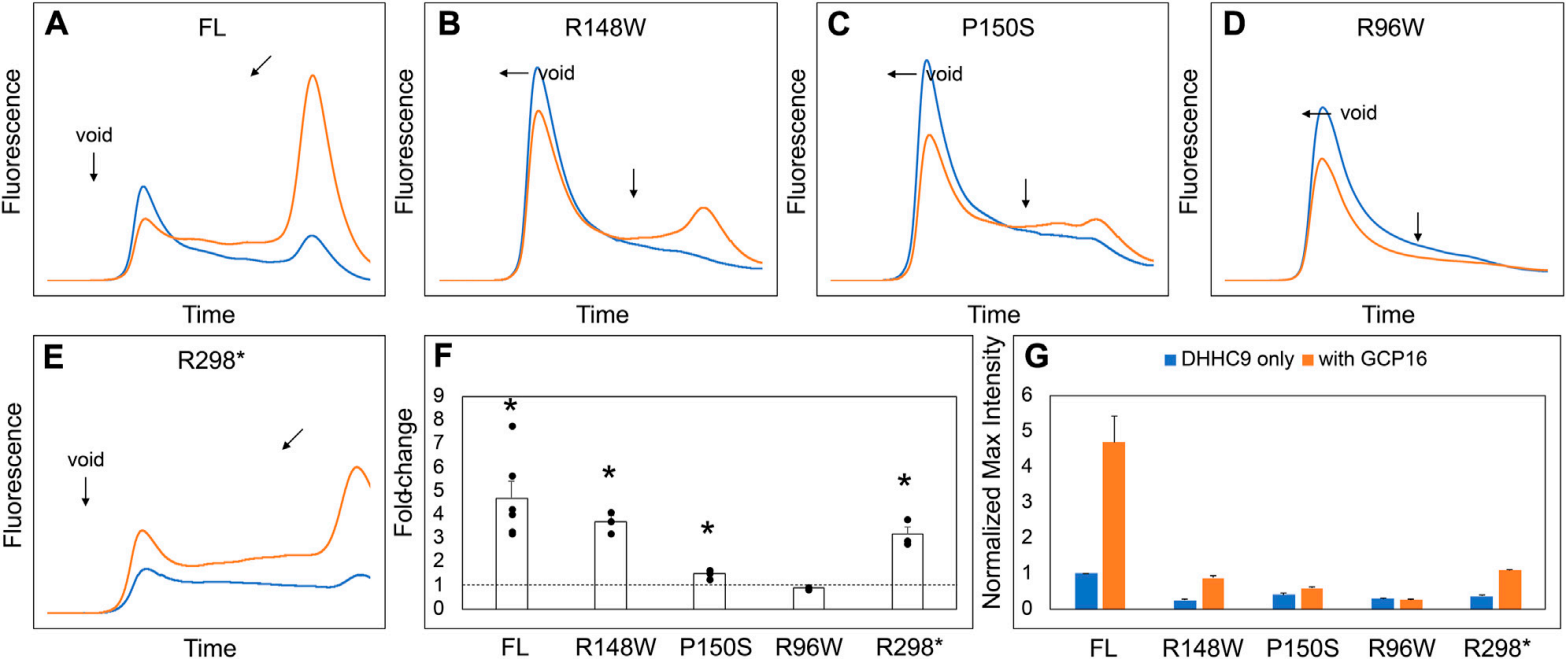
DHHC9 Disease Mutations Exhibit Reduced Protein Stability. **(A–E)** FSEC profiles for full-length DHHC9 (FL), the indicated point mutations, and DHHC9 truncation at R298 (R298*). HEK cells were transfected with vector to express DHHC9 without (blue) and with GCP16 (orange). Bar charts showing **(F)** average fold-change and **(G)** normalized max intensity upon GCP16 co-expression for the indicated constructs at n ≥ 3 experiments. Max intensities were normalized to DHHC9 FL as described in the experimental procedures. The dashed line indicates a fold-change of 1. Error bars represent the standard error of the mean. Asterisks indicate a significance for *p*-value <0.05 determined by two-tailed *t*-test against the null hypothesis.

DHHC9 protein pathogenic variants include missense mutations R148W, P150S, and R96W, and a non-sense mutation terminating at R298 (Schirwani et al., 2018). An earlier study reported that DHHC9 (R148W) and DHHC9 (P150S) possessed reduced steady state levels of autopalmitoylated DHHC9 (Mitchell et al., 2014). Given the importance of GCP16 in stabilizing DHHC9 protein folding with a corresponding effect on enzyme activity, we asked how the missense and non-sense mutations affected complex formation with GCP16. Compared to wildtype DHHC9, DHHC9 with R148W, P150S, R96W, or R298X mutations exhibited a greater extent of protein aggregation and reduced monodispersity as monitored by FSEC (Figures 6A–E, blue traces, F). Co-expression with GCP16 resulted in improved protein behavior compared to the respective DHHC9 constructs alone for all the mutants except R96W, although with a diminished effect compared to wildtype (Figures 6A–E, orange traces, F). These results suggest the DHHC9 mutations associated with XLID result in decreased protein stability and reflect reduced formation of a DHHC9-GCP16 complex.

### GCP16 and GOLGA7B stabilize DHHCs in a subtype-specific manner

**FIGURE 7:**
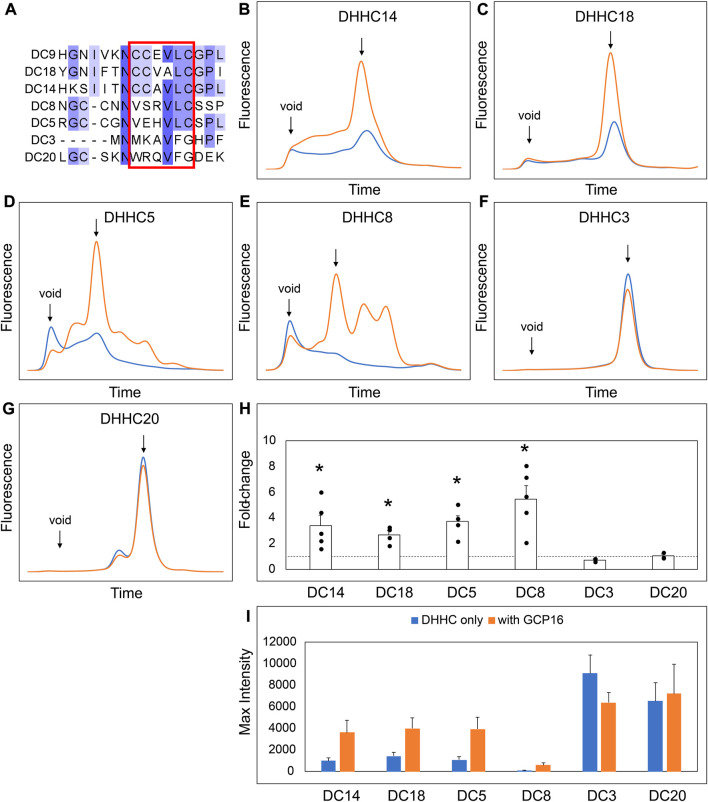
GCP16 Co-expression Stabilizes DHHC Proteins with the Conserved C-terminal Cysteine Motif. **(A)** Amino acid sequence alignment of select DHHC proteins at the conserved CTD cysteine motif. The conserved cysteine motif is boxed in red. **(B–G)** FSEC analysis of crude HEK lystates for the indicated constructs without (blue) or with GCP16 (orange). Bar charts showing **(H)** fold-change upon and **(I)** average max intensity for the indicated constructs upon GCP16 co-expression at n ≥ 3 experiments. Error bars represent the standard error of the mean. Asterisks indicate a significance for *p*-value <0.05 determined by two-tailed *t*-test against the null hypothesis.

**FIGURE 8:**
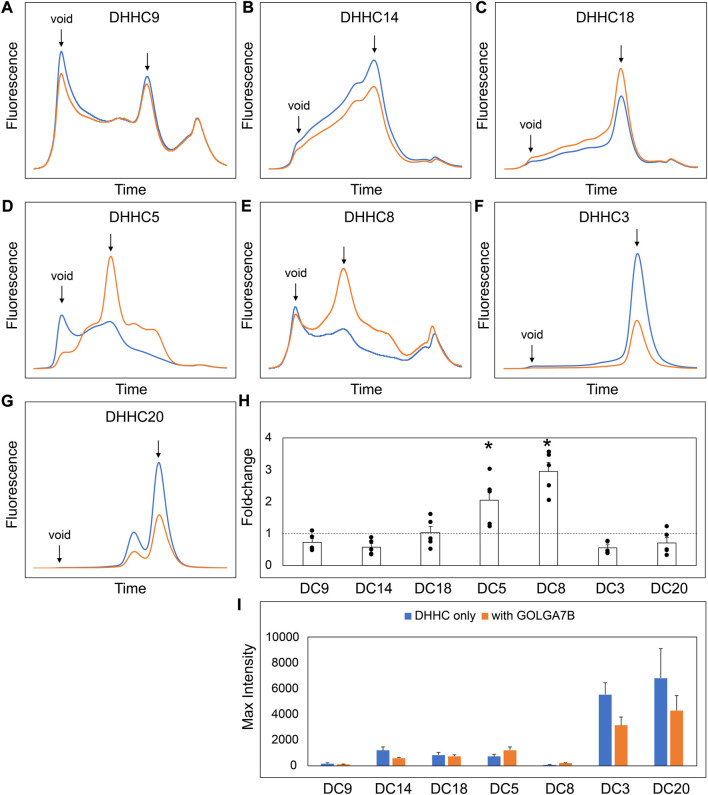
GOLGA7B Co-expression Stabilizes DHHC Proteins. **(A–G)** FSEC profiles for the indicated constructs. HEK cells were transfected with DHHC without (blue) or with GOLGA7B (orange). Bar charts showing **(H)** fold-change upon and **(I)** average max intensity for the indicated constructs upon GOLGA7B co-expression at n = 5 experiments. Error bars represent the standard error of the mean. Asterisks indicate a significant increase with GOLGA7B for *p*-value <0.05 determined by two-tailed *t*-test against the null hypothesis.

Our experiments suggest that the CCM in DHHC9 plays an important role in complex formation with GCP16. This motif is present in other DHHCs closely related to DHHC9, namely, DHHC14, 18, 5, and 8, but it is not present in distantly related DHHCs, such as DHHC3 and DHHC20 (Figure 7A). DHHC9 was the first human DHHC to be identified to require a protein partner for its activation, based on its homology to yeast Erf2-Erf4 (Swarthout et al., 2005). Recent studies suggest that GCP16 and GOLGA7B, a protein with ∼75% amino acid sequence identity to GCP16, function as accessory proteins for additional members of the DHHC protein family. Woodley and Collins reported that DHHC5 interacts with GOLGA7B, facilitating DHHC5 localization at the plasma membrane and enabling its interactions with components of desmosomes to regulate cell adhesion (Woodley and Collins, 2019). Ko et al. identified ZDHHC5 and GOLGA7 (GCP16) in a screen for genes involved in an unconventional non-apoptotic cell death pathway triggered by the synthetic small molecule oxime, CIL56 (Ko et al., 2019). They went on to show that DHHC5 and GCP16 form a mutually stabilizing protein complex localized at the plasma membrane. Complex formation is dependent upon C-terminal cysteines in the conserved CCM motif, consistent with our results for DHHC9-GCP16 complex formation. To determine the potential of other DHHC proteins to form complexes with GCP16 and/or GOLGA7B, we tested whether co-expression of GCP16 or GOLGA7B affects the protein behavior of a set of DHHC proteins using FSEC. Like DHHC9, all DHHCs containing the CCM exhibited improved protein expression and monodispersity when co-expressed with GCP16 (Figures 7B–E,H,I). However, representative DHHCs without the CCM showed no significant difference whether GCP16 was co-expressed (Figures 7F–I). This supports that the GCP16-mediated stabilization is specific to certain DHHCs that possess the CCM. Interestingly, DHHC protein co-expression with GOLGA7B showed a similar, but distinct, result (Figure 8). While GOLGA7B co-expression significantly improved DHHC5 and DHHC8 protein (Figures 8D,E,H,I), it had no significant stabilizing effect on DHHC9, 14, 18, 3, and 20 (Figures 8A–C, F-I). Taken together, our FSEC experiments suggest GCP16 and GOLGA7B stabilize DHHC proteins in a subtype-specific manner.

**FIGURE 9:**
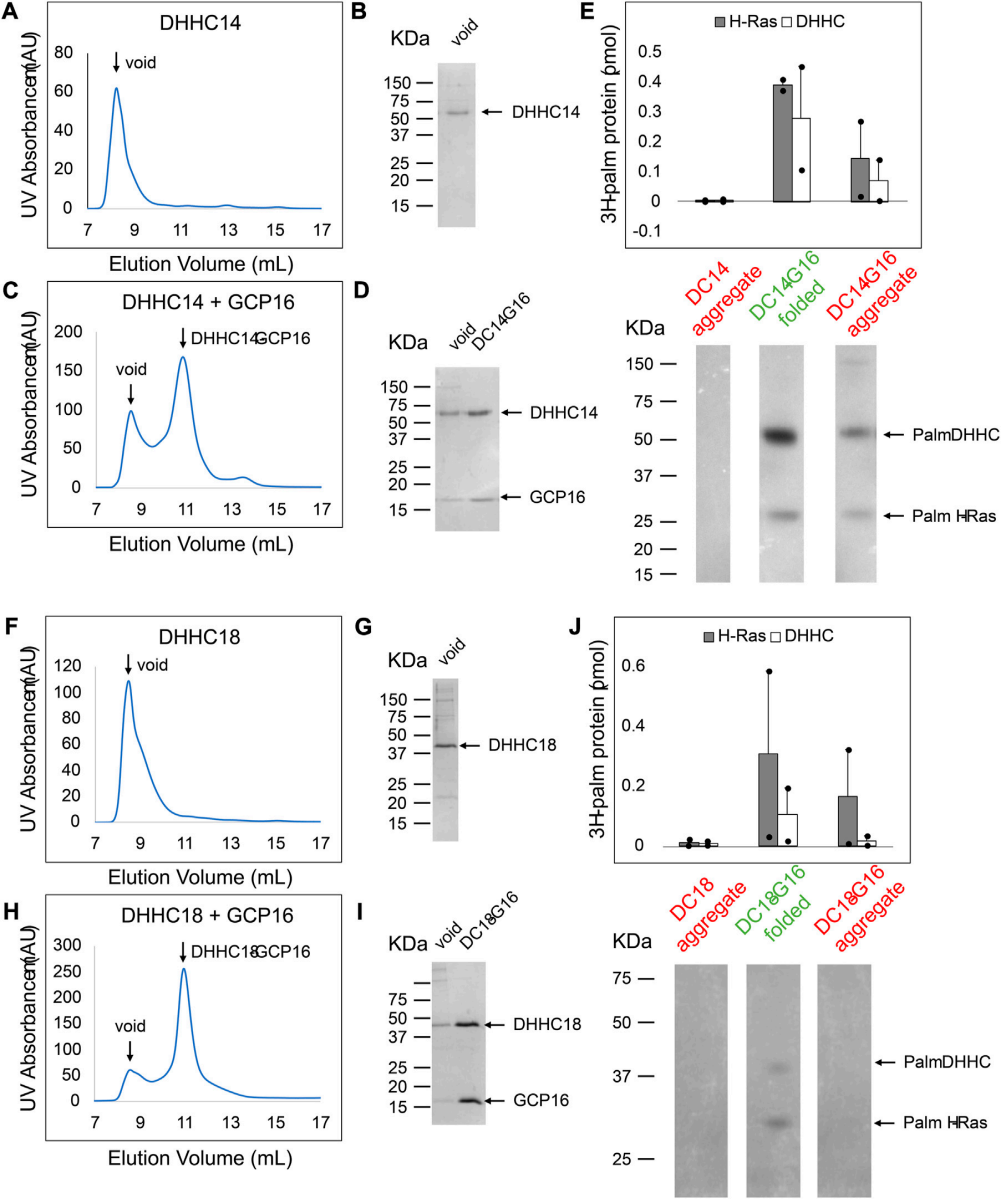
DHHC14 and DHHC18 Each Forms a Complex with GCP16 to Confer PAT Activity. Sf9 cells were infected with recombinant baculovirus encoding strep-tagged DHHC alone or DHHC + GCP16. Protein was purified *via* strep-affinity purification and SEC. SEC profiles and SDS-PAGE Coomassie for **(A,B)** DHHC14 **(C,D)** the DHHC14 co-purified with GCP16, **(F,G)** DHHC18, and **(H,I)** DHHC18 co-purified with GCP16 are shown. Arrows in the SEC profiles correspond to the labeled lanes for the corresponding SDS-PAGE analysis. **(E,J)** Purified DHHC14/DHHC18 was classified into aggregated (red) or folded (green) states based on the SEC profile. DHHC14/DHHC18 was assayed for activity using H-Ras and [^3^H]palmitoyl CoA. Radiolabeled protein was quantified *via* liquid scintillation spectroscopy and detected *via* fluorography. The *y*-axis represents picomoles of ^3^H-labeled protein after subtracting background radiation. Gray bars represent the quantity of labeled H-Ras, and white bars represent the quantity of labeled DHHC protein. Error bars represent the standard error of the mean for n = 2 independent protein preparations. Arrows indicate bands corresponding to radiolabeled DHHC and H-Ras.

To better understand the effect of GCP16 on the DHHC9 subfamily, we purified DHHC14 and DHHC18 and assessed the relationship between protein folding and PAT activity (Figure 9). As was the case with DHHC9, DHHC14 purified without GCP16 resulted in nearly complete protein aggregation (Figures 9A,B) and exhibited no detectable PAT activity (Figure 9E lane 1). On the other hand, DHHC14 co-purified with GCP16 was monodisperse (Figures 9C,D) and the folded complex in the included volume exhibited enzymatic activity for H-Ras and DHHC14 autoacylation (Figure 9E lane 3). Surprisingly, the DHHC14-GCP16 aggregate detected in the void volume exhibited PAT activity, albeit reduced relative to the folded complex (Figure 9E lane 2). Similarly, DHHC18 purified by itself was aggregated and enzymatically inactive (Figures 9F,G,J lane 1). Like DHHC14-GCP16, the DHHC18-GCP16 complex purified as two species with the folded complex having more activity than that found in the aggregate (Figures 9H–J lane 2). These results suggest that the stabilizing effect of GCP16 spans the DHHC9 subfamily.

## Discussion

In this study, we sought to understand the function of GCP16 in the DHHC9-GCP16 complex. We found that co-expression of GCP16 with DHHC9 improved DHHC9 protein levels and homogeneity. Through truncations, mutagenesis, and FSEC screening, we identified a conserved C-terminal cysteine motif in DHHC9 that is required for the GCP16-mediated stabilization effect. We used the SEC profiles of purified proteins to show the CCM is required for formation of the DHHC9-GCP16 complex. Without GCP16, purified DHHC9 aggregates, and only folded DHHC9-GCP16 complex formation is enzymatically active for H-Ras *in vitro*. Furthermore, we showed that disease mutations associated with XLID result in decreased protein stability and diminished DHHC9-GCP16 complex formation.

DHHC9 and GCP16 were discovered based on homology to yeast palmitoyltransferase Erf2-Erf4 (Lobo et al., 2002; Swarthout et al., 2005). DHHC9 and GCP16 share 31% and 17% sequence identity with Erf2 and Erf4, respectively. While the exact sequence identity between the pair is limited, they possess functional similarities. For example, both form heteromeric DHHC protein complexes and are putative Ras PATs in their respective organisms (Lobo et al., 2002; Swarthout et al., 2005). Furthermore, DHHC9-GCP16 can substitute for loss of Erf2 in *S. cerevisiae* (Mitchell et al., 2014). Mitchell et al. showed, in the absence of Erf4, Erf2 steady-state protein levels and its half-life are significantly lowered and that one function of Erf4 is to stabilize Erf2 by impeding its ERAD-mediated degradation (Mitchell et al., 2012). Similarly, our data support that one role for GCP16 is to provide local stability for DHHC9, suggesting this function for the partner protein may be evolutionarily conserved. However, the role of the accessory protein may extend beyond DHHC protein stabilization. For example in yeast, stabilization of Erf2 does not suppress loss of Erf4 *in vivo*, and Erf2 exhibits distinct enzyme kinetics with and without Erf4 *in vitro* (Mitchell et al., 2012). Specifically, the absence of Erf4 does not abolish Erf2 autopalmitoylation, suggesting that residues of Erf4 do not participate directly in this step. However, the absence of Erf4 does increase the rate of hydrolysis of the thioester on the acyl-enzyme intermediate, causing Erf2 to undergo a futile cycle of autopalmitoylation and hydrolysis. Erf4 is required for Erf2 to transfer the palmitoyl group to the protein substrate, with the exact mechanism remaining unclear (Mitchell et al., 2012).

Earlier proteomics studies identified the C-terminal CCX7-13C*(S/T) sequence among DHHC proteins where cysteines centered around the “GCxxN” motif were palmitoylated, and these cysteines in DHHC5 and DHHC8 were palmitoylated *in vivo* (Yang et al., 2010; Collins et al., 2017). Subsequently, Ko et al. showed DHHC5 and GCP16 are mutually stabilizing and that DHHC5-GCP16 complex formation, as assessed by immunoprecipitation, is dependent on the DHHC5 C-terminal cysteines (Ko et al., 2019). The catalytically active DHHC5-GCP16 complex is required for non-apoptotic cell death induced by the synthetic oxime-containing small molecule caspase-independent lethal 56 (CIL56) (Ko et al., 2019). DHHC5 has also been shown to interact with the GCP16 relative, GOLGA7B. Palmitoylation of the DHHC5 C-terminal cysteines controls its interaction with and its ability to palmitoylate GOLGA7B, which in turn regulates DHHC5 internalization and turnover and its protein interactome (Woodley and Collins, 2019). Howie et al. observed that the DHHC5 C-terminus between N218-T334, which includes the CCM, is required for palmitoylation of the Na-pump accessory protein phospholemman (PLM) (Howie et al., 2014). Interestingly, PLM does not bind directly to this region of DHHC5, but rather it associates with DHHC5 through an intermediate, the Na-pump α subunit, which does bind directly to DHHC5 N218-T334 (Plain et al., 2020). Nonetheless, the DHHC5 region containing the CCM is specifically required for palmitoylation of its substrates PLM and Flotillin-2, while other substrates such as PSD-5 and GRIP1 require the PSD-95/Discs-large/ZO-1 homology (PDZ) binding motif (Howie et al., 2014). The CCM we identified in the DHHC9 subfamily overlaps with the end of the previously described CCX7-13C*(S/T) sequence. Taken together, we reason that the CCM cysteines are likely to be palmitoylated and mediate accessory protein interaction either directly or indirectly by exposing a GCP16/GOLGA7B binding site. Interaction with the accessory protein provides protein stability, and it expands the DHHC protein interactome, which may be a mechanism of substrate recruitment and regulation. How subtype preferences for GCP16/GOLGA7B are governed is to be resolved, though it seems likely to involve additional protein domain coordination.

While this manuscript was in revision, a preprint deposited in bioRxiv reported the cryo-EM structures of human DHHC9-GCP16 and yeast Ef2-Erf4 (Yang et al., 2022). Several of their findings are consistent with our results. We found that the CCM motif and in particular, Cys 288, is essential for DHHC9 activity and stability. Yang et al. identified palmitate on Cys288 and showed that its mutation resulted in the loss of catalytic activity (Yang et al., 2022). Within the DHHC9-GCP16 structure, the palmitate attached to Cys288 in the DHHC9 α3 helix inserts adjacent to transmembrane domains 2 and 3 and the α2’ helix of GCP16, thereby promoting membrane association of the DHHC9 α3 helix and adding stability to the DHHC9-GCP16 complex. Noting the conservation of the CCM in DHHC14 and DHHC18, Yang et al. also reported that DHHC14 and DHHC18 formed catalytically active complexes with GCP16 that palmitoylated HRAS and NRAS, corroborating the results reported herein.

Our study demonstrates that a subgroup of the larger DHHC protein family that includes DHHC9, 14, 18, 5, and 8 all require accessory proteins for their stability *in vitro*. Whereas GCP16 potentiated all subfamily members, GOLGA7B potentiated only DHHC5 and DHHC8. Using purified components, we establish that both DHHC14 and DHHC18 when complexed with GCP16 can function as Ras palmitoyltransferases. Our research supports a broader role for GCP16 and GOLGA7B in the function of human DHHC proteins.

## Data Availability

The original contributions presented in the study are included in the article/Supplementary Materials, further inquiries can be directed to the corresponding authors.

## References

[B1] Baker K. Astle D. E. Scerif G. Barnes J. Smith J. Moffat G. (2015). Epilepsy, cognitive deficits and neuroanatomy in males with ZDHHC9 mutations. Ann. Clin. Transl. Neurol. 2, 559–569. 10.1002/acn3.196 26000327 PMC4435709

[B2] Chamberlain L. H. Shipston M. J. (2015). The physiology of protein S-acylation. Physiol. Rev. 95, 341–376. 10.1152/physrev.00032.2014 25834228 PMC4551212

[B3] Collins M. O. Woodley K. T. Choudhary J. S. (2017). Global, site-specific analysis of neuronal protein S-acylation. Sci. Rep. 7, 4683. 10.1038/s41598-017-04580-1 28680068 PMC5498535

[B4] Gonzalez Montoro A. Quiroga R. Valdez Taubas J. (2013). Zinc co-ordination by the DHHC cysteine-rich domain of the palmitoyltransferase Swf1. Biochem. J. 454, 427–435. 10.1042/BJ20121693 23790227

[B5] Gottlieb C. D. Linder M. E. (2017). Structure and function of DHHC protein S-acyltransferases. Biochem. Soc. Trans. 45, 923–928. 10.1042/BST20160304 28630137

[B6] Gottlieb C. D. Zhang S. Linder M. E. (2015). The cysteine-rich domain of the DHHC3 palmitoyltransferase is palmitoylated and contains tightly bound zinc. J. Biol. Chem. 290, 29259–29269. 10.1074/jbc.M115.691147 26487721 PMC4705932

[B7] Han J. Y. Lee I. G. Shin S. Kim M. Jang J. H. Park J. (2017). The first patient with sporadic X-linked intellectual disability with de novo ZDHHC9 mutation identified by targeted next-generation sequencing. Eur. J. Med. Genet. 60, 499–503. 10.1016/j.ejmg.2017.07.002 28687527

[B8] Howie J. Reilly L. Fraser N. J. Vlachaki Walker J. M. Wypijewski K. J. Ashford M. L. (2014). Substrate recognition by the cell surface palmitoyl transferase DHHC5. Proc. Natl. Acad. Sci. U. S. A. 111, 17534–17539. 10.1073/pnas.1413627111 25422474 PMC4267385

[B9] Jennings B. C. Linder M. E. (2012). DHHC protein S-acyltransferases use similar ping-pong kinetic mechanisms but display different acyl-CoA specificities. J. Biol. Chem. 287, 7236–7245. 10.1074/jbc.M111.337246 22247542 PMC3293542

[B10] Kawate T. Gouaux E. (2006). Fluorescence-detection size-exclusion chromatography for precrystallization screening of integral membrane proteins. Structure 14, 673–681. 10.1016/j.str.2006.01.013 16615909

[B11] Ko P. J. Woodrow C. Dubreuil M. M. Martin B. R. Skouta R. Bassik M. C. (2019). A ZDHHC5-GOLGA7 protein acyltransferase complex promotes nonapoptotic cell death. Cell Chem. Biol. 26, 1716–1724 e9. 10.1016/j.chembiol.2019.09.014 31631010

[B12] Lobo S. Greentree W. K. Linder M. E. Deschenes R. J. (2002). Identification of a Ras palmitoyltransferase in *Saccharomyces cerevisiae* . J. Biol. Chem. 277, 41268–41273. 10.1074/jbc.M206573200 12193598

[B13] Malgapo M. I. P. Linder M. E. (2021). Substrate recruitment by zDHHC protein acyltransferases. Open Biol. 11, 210026. 10.1098/rsob.210026 33878949 PMC8059564

[B14] Mitchell D. A. Hamel L. D. Ishizuka K. Mitchell G. Schaefer L. M. Deschenes R. J. (2012). The Erf4 subunit of the yeast Ras palmitoyl acyltransferase is required for stability of the Acyl-Erf2 intermediate and palmitoyl transfer to a Ras2 substrate. J. Biol. Chem. 287, 34337–34348. 10.1074/jbc.M112.379297 22904317 PMC3464540

[B15] Mitchell D. A. Hamel L. D. Reddy K. D. Farh L. Rettew L. M. Sanchez P. R. (2014). Mutations in the X-linked intellectual disability gene, zDHHC9, alter autopalmitoylation activity by distinct mechanisms. J. Biol. Chem. 289, 18582–18592. 10.1074/jbc.M114.567420 24811172 PMC4140262

[B16] Mitchell D. A. Mitchell G. Ling Y. Budde C. Deschenes R. J. (2010). Mutational analysis of *Saccharomyces cerevisiae* Erf2 reveals a two-step reaction mechanism for protein palmitoylation by DHHC enzymes. J. Biol. Chem. 285, 38104–38114. 10.1074/jbc.M110.169102 20851885 PMC2992244

[B17] Mitchell D. A. Vasudevan A. Linder M. E. Deschenes R. J. (2006). Protein palmitoylation by a family of DHHC protein S-acyltransferases. J. lipid Res. 47, 1118–1127. 10.1194/jlr.R600007-JLR200 16582420

[B18] Ohta E. Misumi Y. Sohda M. Fujiwara T. Yano A. Ikehara Y. (2003). Identification and characterization of GCP16, a novel acylated Golgi protein that interacts with GCP170. J. Biol. Chem. 278, 51957–51967. 10.1074/jbc.M310014200 14522980

[B19] Plain F. Howie J. Kennedy J. Brown E. Shattock M. J. Fraser N. J. (2020). Control of protein palmitoylation by regulating substrate recruitment to a zDHHC-protein acyltransferase. Commun. Biol. 3, 411. 10.1038/s42003-020-01145-3 32737405 PMC7395175

[B20] Rana M. S. Kumar P. Lee C. J. Verardi R. Rajashankar K. R. Banerjee A. (2018). Fatty acyl recognition and transfer by an integral membrane S-acyltransferase. Science 359, eaao6326. 10.1126/science.aao6326 29326245 PMC6317078

[B21] Raymond F. L. Tarpey P. S. Edkins S. Tofts C. O'Meara S. Teague J. (2007). Mutations in ZDHHC9, which encodes a palmitoyltransferase of NRAS and HRAS, cause X-linked mental retardation associated with a Marfanoid habitus. Am. J. Hum. Genet. 80, 982–987. 10.1086/513609 17436253 PMC1852737

[B22] Salaun C. Locatelli C. Zmuda F. Cabrera Gonzalez J. Chamberlain L. H. (2020). Accessory proteins of the zDHHC family of S-acylation enzymes. J. Cell Sci. 133, jcs251819. 10.1242/jcs.251819 33203738

[B23] Schirwani S. Wakeling E. Smith K. Study D. D. D. Balasubramanian M. (2018). Expanding the molecular basis and phenotypic spectrum of ZDHHC9-associated X-linked intellectual disability. Am. J. Med. Genet. A 176, 1238–1244. 10.1002/ajmg.a.38683 29681091

[B24] Swarthout J. T. Lobo S. Farh L. Croke M. R. Greentree W. K. Deschenes R. J. (2005). DHHC9 and GCP16 constitute a human protein fatty acyltransferase with specificity for H- and N-Ras. J. Biol. Chem. 280, 31141–31148. 10.1074/jbc.M504113200 16000296

[B25] Woodley K. T. Collins M. O. (2019). S-acylated Golga7b stabilises DHHC5 at the plasma membrane to regulate cell adhesion. EMBO Rep. 20, e47472. 10.15252/embr.201847472 31402609 PMC6776912

[B26] Yang A. Liu S. Zhang Y. Chen J. Feng S. Wu J. (2022). Regulation of RAS palmitoyltransferases by accessory proteins and palmitoylation. bioRxiv. 10.1038/s41594-023-01183-5 38182928

[B27] Yang W. Di Vizio D. Kirchner M. Steen H. Freeman M. R. (2010). Proteome scale characterization of human S-acylated proteins in lipid raft-enriched and non-raft membranes. Mol. Cell Proteomics 9, 54–70. 10.1074/mcp.m800448-mcp200 19801377 PMC2808267

[B28] Yeste-Velasco M. Linder M. E. Lu Y. J. (2015). Protein S-palmitoylation and cancer. Biochim. Biophys. Acta 1856, 107–120. 10.1016/j.bbcan.2015.06.004 26112306

[B29] Young F. B. Butland S. L. Sanders S. S. Sutton L. M. Hayden M. R. (2012). Putting proteins in their place: Palmitoylation in huntington disease and other neuropsychiatric diseases. Prog. Neurobiol. 97, 220–238. 10.1016/j.pneurobio.2011.11.002 22155432

[B30] Zhang M. M. Wu P. Y. Kelly F. D. Nurse P. Hang H. C. (2013). Quantitative control of protein S-palmitoylation regulates meiotic entry in fission yeast. PLoS Biol. 11, e1001597. 10.1371/journal.pbio.1001597 23843742 PMC3699447

