# Supplementary material for: GCP16 stabilizes the DHHC9 subfamily of protein acyltransferases through a conserved C-terminal cysteine motif
Source: Front Physiol. 2023 Mar 23;14:1167094. doi: 10.3389/fphys.2023.1167094 (PMC10076531; doi:10.3389/fphys.2023.1167094)
Supplement: Supplementary file 1 [file DataSheet1.PDF]

Supplementary Material

GCP16 Stabilizes the DHHC9 subfamily of Protein Acyltransferases through a Conserved C-terminal Cysteine Motif

Phillip L. Nguyen, Wendy K. Greentree, Toshimitsu Kawate*, and Maurine E. Linder*

*** Correspondence:** Toshimitsu Kawate: toshi.kawate@cornell.edu; Maurine E. Linder: mel237@cornell.edu

Supplementary Figures

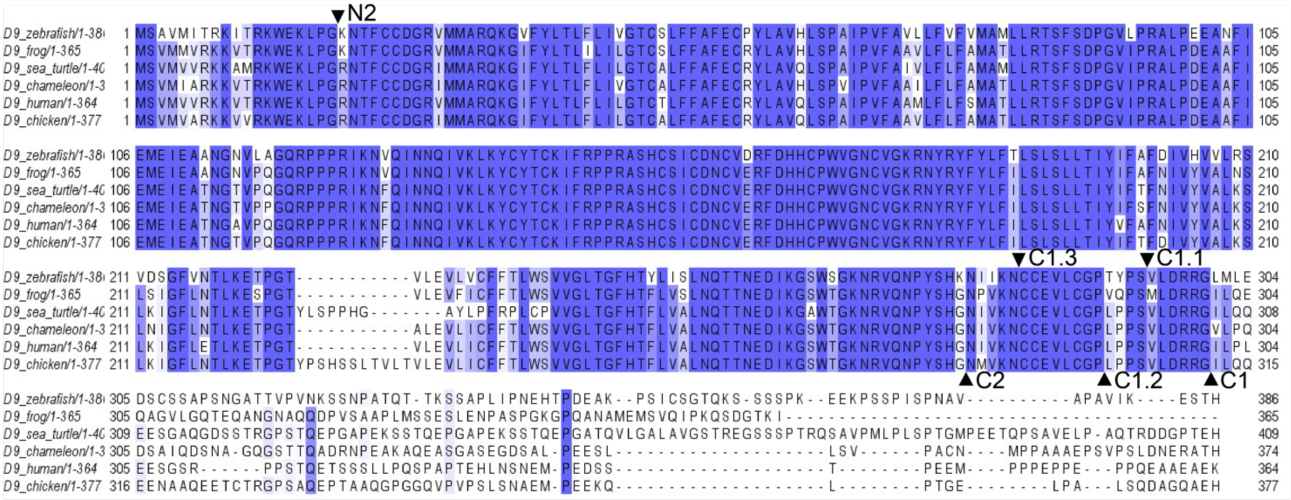

Supplementary Figure 1. Designing DHHC9 Deletion Constructs. Amino acid sequence of human DHHC9 compared to various orthologs. Amino acids highlighted in dark blue are completely conserved, in light blue are somewhat conserved, and in white are not conserved. Arrows indicate where the designated deletions were made.

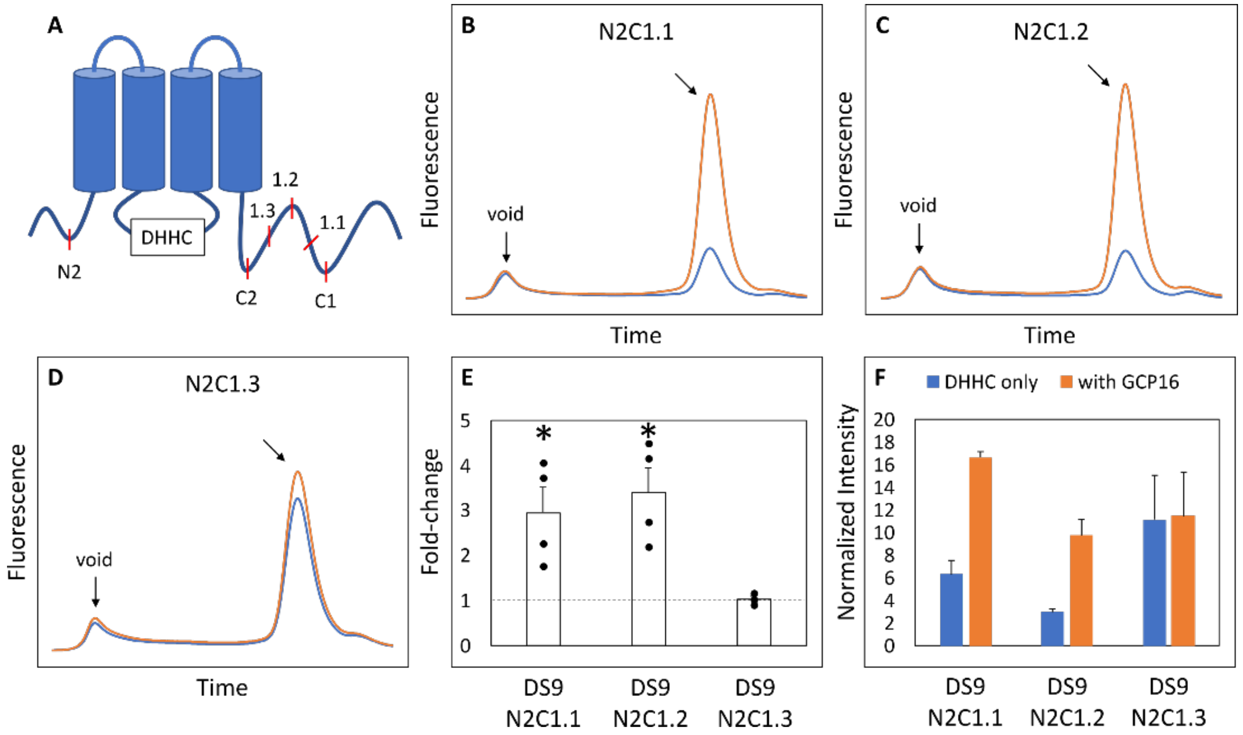

Supplementary Figure 2. Defining the region of the DHHC9 CTD required for GCP16-mediated stabilization. (A) Cartoon depicting the tested DHHC9 truncations. (B-D) FSEC profiles for the indicated constructs. HEK cells were transfected with DHHC9 without (blue) or with GCP16 (orange). Cells were solubilized in DDM-containing buffer, and the cleared lysate was analyzed by FSEC. Bar charts showing (E) average fold-change and (F) normalized max intensity upon GCP16 co-expression for the indicated constructs at $n \geq 3$ experiments. The dashed line indicates a fold-change of 1. Error bars represent the standard error of the mean. Asterisks indicate a significance for p-value <0.05 determined by two-tailed t-test against the null hypothesis.
